# Adaptation of Foxtail Millet (*Setaria italica* L.) to Abiotic Stresses: A Special Perspective of Responses to Nitrogen and Phosphate Limitations

**DOI:** 10.3389/fpls.2020.00187

**Published:** 2020-02-28

**Authors:** Faisal Nadeem, Zeeshan Ahmad, Mahmood Ul Hassan, Ruifeng Wang, Xianmin Diao, Xuexian Li

**Affiliations:** ^1^MOE Key Laboratory of Plant-Soil Interactions, Department of Plant Nutrition, China Agricultural University, Beijing, China; ^2^Institute of Crop Sciences, Chinese Academy of Agricultural Sciences, Beijing, China

**Keywords:** foxtail millet, abiotic stresses, nitrogen limitation, phosphate starvation, transporter

## Abstract

Amongst various environmental constraints, abiotic stresses are increasing the risk of food insecurity worldwide by limiting crop production and disturbing the geographical distribution of food crops. Millets are known to possess unique features of resilience to adverse environments, especially infertile soil conditions, although the underlying mechanisms are yet to be determined. The small diploid genome, short stature, excellent seed production, C_4_ photosynthesis, and short life cycle of foxtail millet make it a very promising model crop for studying nutrient stress responses. Known to be a drought-tolerant crop, it responds to low nitrogen and low phosphate by respective reduction and enhancement of its root system. This special response is quite different from that shown by maize and some other cereals. In contrast to having a smaller root system under low nitrogen, foxtail millet enhances biomass accumulation, facilitating root thickening, presumably for nutrient translocation. The low phosphate response of foxtail millet links to the internal nitrogen status, which tends to act as a signal regulating the expression of nitrogen transporters and hence indicates its inherent connection with nitrogen nutrition. Altogether, the low nitrogen and low phosphate responses of foxtail millet can act as a basis to further determine the underlying molecular mechanisms. Here, we will highlight the abiotic stress responses of foxtail millet with a key note on its low nitrogen and low phosphate adaptive responses in comparison to other crops.

## Introduction

Abiotic and biotic environmental stresses reduce plant growth and yield below optimum levels. According to an FAO report released in 2007, only 3.5% of the global area is not affected by environmental constraints, contributing to 50–70% of crop yield reduction (Boyer, 1982; Mittler, 2006). Being sessile in nature, plants encounter these environmental challenges while obtaining the carbon, water, and nutrients necessary for development, growth, and biomass production. The dynamic and complex responses of plants to abiotic stresses can be elastic (reversible) or plastic (irreversible) (Cramer, 2010; Skirycz and Inze, 2010). Plant growth is based on cell proliferation, which requires the persistent availability of nutrients, water, and energy; hence, plants survive through acclimatory responses to nutrient, water, light, and temperature fluctuations.

Roots are vital for optimum crop production because, as well as their water and nutrient uptake functionality, they provide anchorage of plants to soils, store essential elements, and have symbiotic relationships with microorganisms present in the rhizosphere (Bechtold and Field, 2018). Drought, soil salinity, and nutrient toxicity and deficiency are frequent stresses directly encountered by plant roots, leading them to modify or alter their growth as per environmental signaling. The geochemical processes of rock weathering replenish soils with nutrients, except for nitrogen, which originates primarily from atmospheric nitrogen (N) fixation. The natural impoverishment of some nutrients leads to their deficiencies in soils. Nutrient limitation is a limiting factor in crop growth and production that originates from a combination of natural and anthropogenic processes (Sanchez and Salinas, 1981; Giehl and von Wirén, 2014).

N is an important macronutrient governing crop productivity through the regulation of growth and development. N exists in soils heterogeneously, either as inorganic forms, i.e., nitrate and ammonium, or organic forms, like amino acids, peptides, and lipids. Organic forms of nitrogen persist in specific habitats such as boreal and tropical ecosystems. Nitrate and ammonium are the predominant forms of N in most soils, and their availability is controlled by soil physical properties, leaching, and microbial activities, more often than not resulting in formation of N-depletion zones in soils (Miller and Cramer, 2004; Jones and Kielland, 2012; Werdin-Pfisterer et al., 2012); upon N limitation, plants develop physiological alterations to enhance nitrogen acquisition (Good et al., 2004; Hermans et al., 2006; Nacry et al., 2013) or farmers apply synthetic fertilizers to ensure yield. The latter often leads to the deterioration of soil physical properties on the one hand, whereas it results in N losses through leaching (polluting ground-water reservoirs), runoff (deposition in fresh-water bodies, causing eutrophication), NH_3_-volatilization, and denitrification on the other hand. Excessive N deposition negatively influences air quality and ecosystem health by impacting human health, unbalancing greenhouse gas emissions, disturbing soil and water chemistry, and narrowing biological diversity (Tilman et al., 2006; Guo et al., 2009; Sutton et al., 2011; Stevens et al., 2015; Liu et al., 2016). Hence, to counter (1) environmental risks and economic losses associated with N-fertilization, and (2) the scarcity of N in natural soils, it is worth investigating the morphological, physiological, and molecular adaptive alterations adopted by plants to survive in N-limiting environments.

Phosphorus (P), a key component of nucleic acid and phospholipids, is another macronutrient that is essential for plant growth and development. It exists in soils either as inorganic phosphorus (Pi) interacting strongly with divalent and trivalent cations or as organophosphates releasing phosphorus for root uptake upon hydrolysis. In traditional agricultural systems, farmers either rely on the inherent fertility of the soil or the addition of manures and phosphate fertilizers to supply Pi for plant growth (Syers et al., 2008). However, the acquisition of phosphorus from soils is challenging for plants because of the low solubility of phosphates of aluminum, iron, and calcium (Schachtman et al., 1998). Pi also has high sorption capacity to soil particles; thus, its uptake by plants depends upon their ability to find immobile Pi in soils. Hence, the unavailability of P in soils and agricultural intensification have resulted in a dependency on the application of Pi fertilizers to increase crop yield (Cordell et al., 2009).

Different plants have evolved differential responses to cope with N, P, and other abiotic stresses. Research on the abiotic stress responses of plants has come to the forefront but now needs to be extended beyond maize, rice, wheat or *Arabidopsis thaliana* to enhance crop diversity. Foxtail millet (*Setaria italica* L.), thought to be native of South Asia, is one of the oldest cultivated millets around the globe. The cultivation of foxtail millet for human consumption dates back to 4000 years ago (Baltensperger, 1996). China, Russia, India, Africa, and the United States are the regions where it is widely grown. It is being cultivated for food and fodder throughout Eurasia and the Far East. It is primarily grown for hay in the United States and can produce 2.47–8.65 tones ha^–1^ aboveground biomass (Schonbeck and Morse, 2006). Its C/N ratio is 44, and it contains 48 kg ha^–1^ N in aboveground biomass (Creamer and Baldwin, 1999). It produces high yield with low levels of prussic acid (Sheahan, 2014). In contrast to other millets, foxtail millet can be grown in cooler and droughty regions in spite of having a shallow root system (Hancock Seed, 2014). It is a water-efficient crop: it requires 1/3 less water than maize and can produce one ton of forage in 2½ inch moisture (Koch, 2002). Foxtail millet is also a preferred choice for the restoration of steep slopes or mine lands because it grows fast and produces more biomass than annual rye (Burger et al., 2009).

**FIGURE 1:**
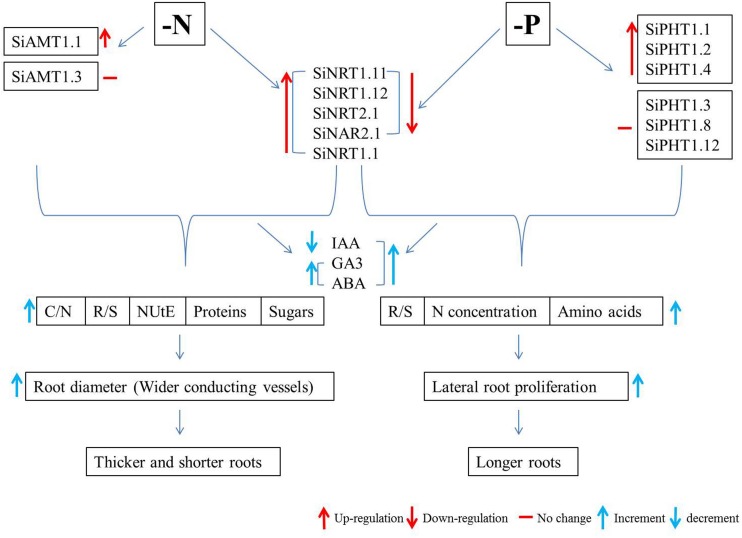
Flow chart of the nitrogen- and phosphorus-limitation responses of foxtail millet. Si, *Sitaria italica*; AMT, ammonium transporter; NRT, nitrate transporter; NAR, nitrate assimilation-related protein; PHT, phosphate transporter; IAA, indole-3-acetic acid; GA3, gibberellic acid; ABA, abscisic acid; C/N, carbon to nitrogen ratio; R/S, root to shoot ratio; NUtE, nitrogen utilization efficiency; N, nitrogen.

Since the release of genome sequences of foxtail millet by the Joint Genome Institute (JGI) of the United States Department of Energy, the importance of this species has been increasingly growing. Owing to its close relationship with bioenergy crops like switch grass (*Panicum virgatum*), Napier grass (*Pennisetum purpureum*), and pearl millet (*Pennisetum glaucum*), foxtail millet is also considered as a model system for biofuel grasses (Doust et al., 2009). Bennetzen et al. (2012) and Zhang et al. (2012) have compiled two full reference genome sequences along with high-density linkage maps with another foxtail millet line and green foxtail and have examined the evolution and mechanisms of C_4_ photosynthesis in foxtail millet. The availability of the foxtail millet genome provides an important resource for studying C_4_ photosynthesis in the context of carbon, N, and P metabolism and nutrient use efficiency. The molecular basis of drought tolerance can also be investigated through drought-associated genes (Zhang et al., 2012). Intensive studies are expected to be conducted on foxtail millet as a model crop for plant nutrient use efficiency, which may benefit agricultural sustainability and food security by enhancing crop diversity (Doebley, 2006). Therefore, the current review will be focusing on the abiotic stress responses of foxtail millet, with special emphasis on its responses to low N and Pi (Figure 1).

## Foxtail Millet: a Model Crop for Stress Biology

Foxtail millet is a herbicide (Zhu et al., 2006) and is a drought and salt-tolerant crop (Jayaraman et al., 2008; Krishnamurthy et al., 2014; Sudhakar et al., 2015). Foxtail millet produces 1 g of dry mass at the cost of 257 g of water, which is far less than that required by maize and wheat (Li and Brutnell, 2011). Auxin response factors (ARFs) regulate embryogenesis, leaf expansion and senescence, and lateral root and fruit development by controlling the expression of auxin response genes (Wilmoth et al., 2005). Since ARF1 isolation (Regad et al., 1993), ARFs have been identified in plant species like *Arabidopsis*, rice, tomato, potato, maize, carrot, wheat, tobacco, and barley. In maize, ARF proteins are involved in the transformation of lipid composition indirectly (Verwoert et al., 1995), and overexpression of *ZmARF1* in *Arabidopsis* enhances growth rates by increasing leaf and seed size (Yuan et al., 2013). Similarly, overexpression of *ZmARF2* in *Arabidopsis* produces larger leaves and seeds and taller plants due to enhanced cell expansion (Wang et al., 2012). ARF proteins play roles in biotic and abiotic stress tolerance in crop plants. Identification of ARF/ARL gene family members in foxtail millet and rice, together with characterization of their structure, organization, duplication and divergence and expression patterns in different tissues, has been reported (Muthamilarasan et al., 2016). A total of 25 ARF genes were identified in foxtail millet diverged from a common ancestor. More efforts are required to investigate ARF genes in specific tissue under a specific stress condition to gain clear clues on tissue-specific and/or stress-inducible promoters. WRKYs are one of the largest transcription factor families and contain W-box in their promoter region to control gene expression and regulation in plants (Eulgem et al., 2000). Comprehensive computational approaches have also been used to identify WRKY genes in foxtail millet. Differential expression patterns of candidate *SiWRKY* genes under abiotic stresses suggest their stress-related regulatory functions (Muthamilarasan et al., 2015).

Foxtail millet responds to abiotic stresses through enhanced biochemical activities like higher levels of antioxidants, reactive oxygen species, and their scavenging enzymes, enzyme activities of catalase and superoxides, and synthesis of osmolytes and their stress-related proteins (Lata et al., 2011b). Aldo-Keto reductases (AKRs) are known to be cytosolic, monomeric oxidoreductases catalyzing NADPH-dependent reduction activities on carbonyl metabolites (Bohren et al., 1989). A broad range of substrates like sugars, prostaglandins, chalcones, aliphatic/aromatic aldehydes, and some toxins can be metabolized by AKRs (Narawongsanont et al., 2012). AKRs are also known for their effective detoxification of reactive carbonyls produced during severe oxidative stress. AKR (*MsALR*) proteins in transgenic tobacco plants improve tolerance against methylviologen, heavy metals, osmotic stress, and long periods of oxidative stresses induced by drought (Oberschall et al., 2000), cold (Hegedus et al., 2004), and UV radiation (Hideg et al., 2003). In tobacco plants, heterologous expression of *OsAKR1* shows better tolerance against heat (Turoczy et al., 2011). Overexpression of *Arabidopsis* AKR4C9 in barley enhances freezing tolerance and post-frost regenerative capacity (Eva et al., 2014). Moreover, overexpression of peach AKR1 (*PpAKR1*) in *Arabidopsis* enhances salt tolerance compared to wild type plants (Kanayama et al., 2014). In contrast, *GmAKR1* protein overproduction inhibits nodule development in the hairy roots of soybean (Hur et al., 2009). Malondialdehyde (MDA), a product of lipid peroxidation, is a biomarker of oxidative stresses (Bailly et al., 1996); lower MDA levels indicate better oxidative stress tolerance. *OsAKR1* overexpression in tobacco lowers levels of MDA and methylglyoxal (MG) in leaf tissues under chemical and heat stress treatments (Turoczy et al., 2011). Foxtail millet *AKR1* is a promising stress-responsive gene that modulates and enhances stress tolerance in major crops (Kirankumar et al., 2016). Thus, investigation of the functions of AKR in reactive carbonyl detoxification and the promotion of abiotic stress tolerance in foxtail millet is of interest.

Reactive oxygen species (ROS) are involved in various signal transduction pathways (Apel and Hirt, 2004; Laloi et al., 2004) under stress conditions (Mustilli et al., 2002; Neill et al., 2002). ROS also regulate gene expression under N, P, and potassium deficiency (Shin and Schachtman, 2004; Shin et al., 2005). Superoxide dismutase converts O_2_^–^, an important component of ROS, into H_2_O_2_ (Fridovich, 1997). Several classical peroxidases like ascorbate peroxidase (APX), glutathione peroxidases, and catalase (CAT) quench the resulting H_2_O_2_. APX and glutathione reductase (GR) detoxify H_2_O_2_ in green leaves (Sofo et al., 2015). They likely act as dehydration stress-responsive components in foxtail millet (Lata et al., 2011b). Maintenance of membrane stability, relative water content, higher levels of APX, CAT, and GR activities, and lower levels of lipid peroxidation and electrolyte provides resistance against the drought stress in foxtail millet (Lata et al., 2011b). Upregulation of phospholipid hydroperoxide glutathione peroxidase (PHGPX) in salt-tolerant foxtail millet lines suggests its role in salt resistance (Sreenivasulu et al., 2004). Aldose reductase is involved in sorbitol biosynthesis and the detoxification of 4-hydroxynon-2-enal (a lipid peroxidation product) in foxtail millet under salt stress; glutathione S-transferase also catalyzes 4-hydroxynon-2-enal detoxification under stress conditions (Veeranagamallaiah et al., 2009). Differentially expressed ESTs and peptides between salt-tolerant and sensitive cultivars (Puranik et al., 2011a), along with other proteins involved in the NaCl stress (Veeranagamallaiah et al., 2008), can be extended to future studies in foxtail millet.

APETALA 2/ethylene responsive element binding factor (AP2/ERF) superfamily members contain a characteristic conserved AP2 domain to bind the core DRE (Dehydration Responsive Element) (5′-A/GCCGAC-3′) *cis*-acting element in the promoter region of target genes (Yamaguchi-Shinozaki and Shinozaki, 1994; Yamaguchi-Shinozaki and Shinozaki, 2006). The single nucleotide polymorphism (SNP) of a dehydration-responsive element binding (DREB) gene is associated with stress tolerance (Lata et al., 2011a). A similar SNP accounts for 27% of variations in stress-induced lipid peroxidation in foxtail millet (Lata and Prasad, 2013). Re-sequencing of foxtail millet may identify vast libraries of SNPs and other markers (Bai et al., 2013; Jia et al., 2013). Small interfering RNAs and non-coding RNAs also have their regulatory roles in drought responses in foxtail millet (Qi et al., 2013). In addition, late embryogenesis-abundant proteins protect higher plants against environmental stresses; *SiLEA14* plays an important role in resisting abiotic damage in foxtail millet (Wang et al., 2014). Its small genome (**∼**490 Mbp; Bennetzen et al., 2012; Zhang et al., 2012) and a wide array of stress responses make foxtail millet a model cereal crop for stress biology and functional genomics (Table 1).

**TABLE 1:** Genes functionally characterized in foxtail millet.

Gene	Functions	References
SET domain genes	Abiotic stress tolerance	Yadav et al., 2016
PHT1 gene family	Phosphate transporters	Ceasar et al., 2014
Argonaute protein 1 encoding gene	Regulation of stress responses	Liu et al., 2016
Abscisic acid stress ripening gene (ASR)	Tolerance to drought and oxidative stresses	Feng et al., 2016
Autophagy-related gene (ATG)	Tolerance to nitrogen starvation and drought stresses	Li et al., 2015
Late embryogenesis abundant protein (LEA)	Tolerance to salt, osmotic, and drought stresses	Wang et al., 2014
ABA-responsive DRE-binding protein (ARDP)	Tolerance to salt and drought stresses	Li et al., 2014
WD-40	Associated with dehydration stress-responsive pathway	Mishra et al., 2012
Acetyl-CoA carboxylase	Resistance to sethoxydim herbicide	Dong et al., 2011
Dehydration-responsive element-binding protein 2 (DREB2)	Dehydration tolerance	Lata et al., 2011a
NAC transcription factor	Salinity tolerance	Puranik et al., 2011b
Si69	Aluminum tolerance	Zhao et al., 2009
Aldose reductase	Associated with salinity stress-responsive pathway	Veeranagamallaiah et al., 2009
Glutamine synthetase Pyrroline-5-carboxylate reductase		Veeranagamallaiah et al., 2007
12-oxophytodienoic acid reductase (OPR1)	Drought tolerance	Zhang et al., 2007
Photosystem II D1protein	Atrazine resistance	Jia et al., 2007
Phospholipid hydroperoxide glutathione peroxidase (*PHGPX*)	Associated with salinity tolerance	Sreenivasulu et al., 2004
Nuclear factor-Y (*SiNF-YA1, SiNFYB8*) genes	Drought and salt tolerance	Feng et al., 2015
Nitrate transporters (*SiNRT*), Ammonium transporters (*SiAMT*)	Nitrate and ammonium uptake and transport	Nadeem et al., 2018
Phosphate transporters (*SiPHP*)	Phosphate transport	Ahmad et al., 2018

## Responses of Foxtail Millet to N Limitation

Roots are the means by which plants take up nutrients; hence, root architectural modifications become vital to explore N under its low availability. Different crop species respond to external low-N conditions differentially. Legumes, for example, develop root nodules to capture atmospheric N through N-fixation (Postgate, 1998), whereas cereals such as maize enhance their root surface area by means of increasing axial and lateral root length to access N in a heterogeneous environment (Wang et al., 2003; Chun et al., 2005). As mentioned before, plants undergo these morphological and physiological alterations to maximize their N use efficiency (NUE), which can be discussed as either N utilization efficiency (NUtE) or N uptake (acquisition) efficiency (Garnett et al., 2009; Xu et al., 2012; Wang et al., 2019). At one extreme, the carbon to N ratio and biomass accumulation (dry weight; root to shoot ratio) in roots of foxtail millet increase under low N, which suggests that its higher N utilization efficiency contributes to maximize its N use efficiency, whereas on the other extreme, foxtail millet responds to increase N translocation efficiency by root thickening. These adaptive responses of foxtail millet to low N signals along with regulation of N uptake activities through N influx transporters located at the plasma membrane eventually maximize N acquisition efficiency (Nadeem et al., 2018).

Surprisingly, foxtail millet produces a specific root length (SRL) of 46852 cm g^–1^ of root dry weight under low N (Nadeem et al., 2018), which is 10 times that of maize seedlings under similar conditions (Han et al., 2015). In addition to the SRL, the average root diameter of low-N foxtail millet also increases (Nadeem et al., 2018). Resource absorption and transportation are two important resource acquisition processes for roots, with the former being used by cortex and the latter by stele (Guo et al., 2008). The ratio of cortex to stele thickness determines the suitability of a plant species for adapting to a certain environment for favorable resource distribution. The increased thickness of foxtail millet roots under low N indicates the anatomical modification of stele, where it can accommodate more conduits like vessels and tracheid for efficient transport of N and metabolites.

Hormones help plants to adapt to environmental cues through the regulation of growth and development (Wolters and Jürgens, 2009; Marsch-Martinez and de Folter, 2016). Indole-3-acetic acid (IAA) regulates primary and lateral root growth (Sabatini et al., 1999; Casimiro et al., 2001), whereas cytokinins (CKs) influence apical root dominance (Aloni et al., 2006). IAA and CK accumulation decreases during root shortening in foxtail millet under low N despite enhanced carbon allocation toward the roots (higher dry mass and C/N ratio). Contrary to IAA and CKs, gibberellic acid (GA3) concentrations increase in the root and shoot of foxtail millet (Nadeem et al., 2018). Accumulation of GA3 antagonistic to IAA and CKs could have contributed to root thickening (increased root diameter) through tissue differentiation and anatomical modifications to roots (Yamaguchi, 2008). Abscisic acid (ABA) is known to be an internal signal of stress responses (Wilkinson and Davies, 2002; Kiba et al., 2011). Higher levels of ABA in N-deprived roots (Nadeem et al., 2018) is rather a stress response of foxtail millet that needs to be further dissected to determine the underlying mechanisms.

Sensing the external nutritional alterations, certain specific proteins act as channels, pumps, or transporters in roots to acquire nutrients from their vicinity and transport them within the root or along the vasculature for long-distance source-to-sink transport (Tegeder and Masclaux-Daubresse, 2018). To transport nitrate (NO_3_^–^), a nutrient as well as a signaling molecule for plant growth and root system modifications (Vidal and Gutiérrez, 2008; Krouk et al., 2010; Alvarez et al., 2012), plants have evolved a high-affinity transport system (HATS) and low-affinity transport system (LATS) (Crawford and Glass, 1998). *NRT2.1* belongs to the high-affinity nitrate transport system (Li et al., 2007), whereas *NRT1.1* is a sensor as well as a dual-affinity nitrate transporter (transceptor) in *Arabidopsis* (Tsay et al., 1993; Liu and Tsay, 2003). NRT2 transporters interact with nitrate accessory protein *NAR2.1* (NAR2 like-proteins) for nitrate absorption. Upregulation of expressions of *SiNRT1.1*, *SiNRT2.1*, and *SiNAR2.1*, together with root architectural modifications, optimizes N acquisition in foxtail millet, which is confirmed by enhanced ^15^N influx into roots (Nadeem et al., 2018). Once nitrate is absorbed, the next phase is its redistribution or translocation from root to shoot and from mature expanded leaves to the youngest leaves (Okamoto et al., 2006; Orsel et al., 2006; Tsay et al., 2007; Miller et al., 2009). In this regard, *NRT1.11* and *NRT1.12* mediate xylem to phloem loading and redistribution of nitrate in *Arabidopsis* (*Arabidopsis thaliana*) leaves with normal nitrate provision (Hsu and Tsay, 2013). Foxtail millet seedlings supplied with only 0.02 mM of NH_4_NO_3_ for 7 days show nitrate redistribution in the shoot through upregulation of *SiNRT1.11* and *SiNRT1.12* expressions, indicating an extraordinary ability to adapt to extreme N limitation. Alongside nitrate uptake, ammonium uptake and transport are controlled by ammonium transporters (AMTs) (Loqué and von Wirén, 2004). *SiAMT1.1* accelerates N acquisition by upregulating its expression (Nadeem et al., 2018).

Interlinked carbon and N metabolism generally give rise to balanced carbohydrates to N-metabolites ratios in plant tissues. However, N limitation leads to higher carbon/N ratios (Sun et al., 2002; Reich et al., 2006; Taub and Wang, 2008). In foxtail millet, the root and shoot maintain the balance between free amino acids and total soluble sugar concentrations owing to low N concentrations of these tissues under low external N provision. Interestingly, concentrations of total soluble proteins in roots increase, in contrast to those in shoots (Nadeem et al., 2018), indicating the probable role of proteins (enzymes in particular) in the regulation of carbon and N metabolism-related cellular activities at the tissue level.

## Foxtail Millet Responses to Pi Starvation

Plants have evolved strategies for enhancing their P-uptake capacity either through arbuscular mycorrhizal symbiosis or modification of the root system architecture (Marschner, 1995; Lambers et al., 2008; Cheng et al., 2011). As explained previously, root system modification is the primary adaptive strategy of plants coping with low availability of nutrients. Low mobility of Pi in soils favors shallow root plants (Panigrahy et al., 2009; Péret et al., 2011; Li et al., 2012; Shi et al., 2013). In *Arabidopsis thaliana*, reduced Pi metabolism (Nussaume et al., 2011; Wang et al., 2011), indirect low-P-mediated stress effects (Thibaud et al., 2010), and genetic control of root responses to low P (Svistoonoff et al., 2007; Ticconi et al., 2009) inhibit primary root growth (Abel, 2011; Niu et al., 2013; Giehl et al., 2014). In addition, blue light suppresses elongation of the primary root of petri dish-grown *Arabidopsis* seedlings under Pi deficiency (Zheng et al., 2019). Thus, environmental factors collectively reshape root structure in response to Pi starvation.

Plant responses to Pi starvation could also be genotype-dependent (Reymond et al., 2006). In monocots like rice and barley, low Pi has a less pronounced effect on primary root growth, perhaps due to high P reserves in seeds (Calderón-Vázquez et al., 2011), whereas primary root growth of maize is stimulated under low Pi (Li et al., 2012). In contrast with the primary root inhibition, lateral root formation in plants is enhanced by low Pi (Williamson et al., 2001; Hodge, 2004; Pérez-Torres et al., 2008). Foxtail millet, on the other hand, develops a larger root system in terms of crown root length and lateral root number, length, and density under Pi deficiency (Ahmad et al., 2018), which is in total contrast to what is observed under low N (Nadeem et al., 2018). The enlargement of the root system in response to low Pi and reduction under low N in foxtail millet could be due to the immobile and mobile nature of Pi and nitrate, respectively; longer roots can reach immobile Pi at its location, and shorter roots can intercept mobile nitrate in the microenvironment of the rhizosphere. This root enlargement of foxtail millet couples with hormonal enhancements (auxin and GA3) and increase in root to shoot ratio due to the allocation of carbon to the P-deficient root.

A larger root system functions to enhance Pi acquisition by transporters (Rausch and Bucher, 2002). Pi transporters are mostly conserved across cereal crops (Rakshit and Ganapathy, 2014). Pi limitation stimulates transcription of PHT1 members (Mudge et al., 2002; Rae et al., 2003) and induces *OsPHT1.2* expression in the stele and lateral roots, along with upregulation of *OsPHT1.4*, probably to improve Pi uptake through roots and translocation to the shoot (Ye et al., 2015; Zhang et al., 2015). Substantial upregulation of *SiPHT1.1, SiPHT1.2*, and *SiPHT1.4* expression in root tissues possibly preconditions for enhanced Pi uptake to replenish the internal P-reserves, whereas down-regulation of *SiPHT1.3* expression probably assists with the retention of Pi in the shoot (Ahmad et al., 2018). On the other end, respective down-regulation of expression of *SiNRT2.1* and *SiNAR2.1* (in roots) and that of *SiNRT1.11* and *SiNRT1.12* (in shoots) (Ahmad et al., 2018) helps balance the N/P ratio within permissible limits for proper root and shoot functionality (Aerts and Chapin, 2000). Such correlative interpretation of the expression of *SiPHRs* in relation to Pi uptake and source-sink remobilization under low Pi calls for in-depth mechanistic dissection (Jia et al., 2011; Ceasar et al., 2014). Alternatively, foxtail millet uses internal Pi reserves for higher utilization efficiency (Rose et al., 2011).

Interestingly, the reduction of SPAD values in the foxtail millet shoot is in contrast to differential accumulation of free amino acids (higher in shoot and root) and total soluble proteins (lower in shoot and root) under low Pi provision (Ahmad et al., 2018). These variations in the accumulation of N-metabolites upon low P suggest a potential link between N and P nutrition at the physiological level. Nutrient provision affects biomass allocation within plants (Poorter et al., 2012). N and P are both considered as the limiting factors in plant growth and development; therefore, the N/P ratio plays a critical role in resource distribution (Graham and Mendelssohn, 2016). The uptake of N and P is adjusted by whole-plant signaling to balance N/P ratios between plant tissues (Imsande and Touraine, 1994; Raghotama, 1999; Forde, 2002). To maintain the N/P ratio, foxtail millet reduces N translocation toward the shoot under Pi limitation (Ahmad et al., 2018), similar to the terrestrial plants that adapt to low-N conditions by decreasing Pi uptake (Aerts and Chapin, 2000). P and N signals are indeed integrated by nitrate-inducible GARP-type transcriptional repressor 1 (*NIGT1*) in *Arabidopsis*; PHR1 promotes the expression of NIGT1-clade genes under low P, which in turn down-regulates *NRT2.1* expression to reduce N uptake (Maeda et al., 2018). *NIGT1* expression is stimulated when N availability is high in order to repress N starvation genes (Kiba et al., 2018). *NIGT1* also regulates Pi starvation responses by directly repressing expression of Pi starvation-responsive genes and *NRT2.1* to equilibrate N and P (Maeda et al., 2018). The potential involvement of the *PHR1-NIGT1-NRT2.1* pathway in low P responses of foxtail millet and subsequent readouts needs to be further studied in future.

## Conclusion

Foxtail millet has been studied for its structural and functional genomics for the purpose of developing genetic and genomic resources and delineating the physiology and molecular biology of stress tolerance, especially drought and salinity stress tolerance. Apart from its adaptation to drought and salinity, foxtail millet is also an N- and P-efficient crop. In parallel to those in major cereals, studies should be conducted to develop nutrient-efficient and environment friendly cultivars of foxtail millet. Its small genome size, short life cycle and inbreeding nature make foxtail millet a perfect choice for a model crop for studies of a broad range of plant nutritional biology research. This review presents a unique perspective of the adaptation of foxtail millet to low N and low P along with a brief background on various abiotic stress tolerance strategies. It can serve as a base to plan future studies in the field of plant nutritional genomics using foxtail millet as a model crop.

## Author Contributions

FN, ZA, XD, and XL conceived and wrote the review. FN, MU, RW, XD, and XL revised the manuscript. All authors have approved the final manuscript.

## Conflict of Interest

The authors declare that the research was conducted in the absence of any commercial or financial relationships that could be construed as a potential conflict of interest.
